# Randomized controlled trial on healthy volunteers of pharmacokinetic and antimicrobial activity of a novel hydrogel-containing chlorhexidine dressing to prevent catheter-related bloodstream infection

**DOI:** 10.3389/fmed.2023.1335364

**Published:** 2024-01-08

**Authors:** Emili Masferrer, Laura Riera-Rodríguez, Víctor Farré-Alins, Sandra Vilà de Muga, Francisco-Javier Arroyo-Muñoz, María-Dolores González-Caro

**Affiliations:** ^1^Department of Dermatology, Hospital Universitari Mútua Terrassa, Terrassa, Spain; ^2^Iberhospitex, S.A., Barcelona, Spain; ^3^Intensive Care Unit, Hospital Universitario Virgen Macarena, Sevilla, Spain

**Keywords:** dressings, chlorhexidine, hydrogel, catheter-related bloodstream infection, antimicrobial activity

## Abstract

**Introduction:**

Catheter-related blood stream infection (CRBSI) is one of the most relevant complications associated to the use of intravascular catheters. In this context, chlorhexidine gluconate (CHG) releasing dressings have been developed to reduce the catheter colonization rate and the risk of infection. The aim of this study is to analyze the release rate of CHG and the antimicrobial activity of a novel CHG-releasing dressing, Oper film^®^ protect CHG, and to compare these parameters to those of the dressing Tegaderm™ CHG in healthy volunteers.

**Methods:**

The study was performed in a cohort of 25 healthy volunteers. Two commercially available chlorhexidine-containing dressings were evaluated and compared in this study, Oper film^®^ protect CHG and Tegaderm™ CHG. The release of CHG and the antimicrobial capacity was determined for one week.

**Results:**

HPLC analysis revealed that both dressings have an equivalent CHG release to the skin 2 days (Oper film^®^ protect CHG, 321 μg/cm^2^; Tegaderm™ CHG, 279 μg/cm^2^) and 7 days (Oper film^®^ protect CHG, 456 μg/cm^2^; Tegaderm™ CHG, 381 μg/cm^2^) after the placement of the products in the non-disinfected back of the subjects. On the other hand, Oper film^®^ protect CHG and Tegaderm™ CHG similarly reduced colony forming units (CFU) in cultures obtained from the skin under the CHG-containing hydrogel compared to control cultures at both 2 days (control, 3.34 log_10_ cfu/cm^2^; Oper film^®^ protect CHG, 0.64 log_10_ cfu/cm^2^; Tegaderm™ CHG, 0.7 log_10_ cfu/cm^2^) and 7 days (control, 3.95 log_10_ cfu/cm^2^; Oper film^®^ protect CHG, 0.11 log_10_ cfu/cm^2^; Tegaderm™ CHG, 1 log_10_ cfu/cm^2^).

**Discussion:**

Data confirm that the recent commercially available dressing Oper film^®^ protect CHG maintains the release of CHG and the antimicrobial activity during at least 7 days, and possesses equivalent drug release and antimicrobial action to Tegaderm™ CHG.

## 1 Introduction

The use of intravascular (IV) catheters is associated with the risk to develop catheter-related blood stream infection (CRBSI). CRBSI is a serious medical problem linked to an increase of morbidity, mortality, length of hospital stays and healthcare costs (1, 2). In Europe, 1,247 Intensive Care Units (ICUs) of 15 countries were studied during the period 2008–2012. The incidence of primary bacteriaemia in patients with a catheter inserted for more than 48 h was 3.5%. During the same period, it was estimated that 4,505 deaths were a direct consequence of bacteriaemia; furthermore, it was related with an increase of the length stay in ICU of 1.26 million of days (3). In a similar cohort study, it was detected an increase of mortality in patients admitted at ICU that suffered CRBSI (4). Additionally, Zimlichlman et al. analyzed the costs of the most frequent nosocomial infections in USA, being CRBSI the one that had a higher impact in the sanitary system (5).

It has been described that biofilms are the predominant mode of growth in nearly all bacterial species, and they are linked to the occurrence of nosocomial infections arising from catheter insertions. Gram-positive and negative bacteria and yeasts are the main CRBSI-related microorganisms, being *Staphylococcus* spp., *E. coli*, *P. aeruginosa*, *K. pneumoniae* and *S. epidermidis* the most common species reported in the bibliography (6).

Recently, hospitals have made considerable efforts to reduce CRBSI. However, despite the improvements, this type of IV catheters-related complication is still a hospital problem. Data from 2019 show a rate between 0.5 and 5.5 CRBSI per 1,000 catheter days in European ICUs (7). As a considerable number of CRBSI could be prevented (8), current attempts are focused on the development of preventive strategies. For this reason, chlorhexidine-containing dressings have emerged as a promising tool to prevent CRBSI.

Chlorhexidine gluconate (CHG) is an antiseptic drug with a broad spectrum of antimicrobial activity. CHG is lipophilic and positively charged, properties that allow the interaction of the drug with lipopolysaccharides and phospholipids of the bacterial cell wall or the outer membrane. At low concentrations, this contact damages the cell wall, enabling the leakage of low molecular weight components and inhibiting enzymes related to the cytoplasmatic membrane. At high concentrations, CHG penetrates the cell and generates severe intracellular damage that leads to cell death (9).

The incorporation of CHG into catheter dressings decreases the microbial burden on the skin and the catheter colonization by microorganisms could be reduced. It is effective against the most common bacteria that generate CRBSI as well as less frequent pathogens (the most common microorganisms isolated in CRBSI are >30% coagulase-negative *Staphylococcus*, 22% *S. aureus*, 8% *Enterecoccus* and 8% *Candida*) (10).

Moreover, CHG-impregnated dressings have a low risk for the development of antimicrobial resistance since CHG is applied topically, has non-specific mechanisms of actions and is not susceptible to efflux pumps (10). Two studies did not find an association between CHG dressings and CHG resistance (11, 12). Other studies found an increased average resistance to CHG in some bacterial species in *in vitro* assays, although after several decades the variation was low. However, the clinical relevance of these results is very limited since CHG concentrations used in clinical practice are far superior to the minimal inhibitory concentration for any analyzed microbes (9, 13).

Indeed, the updated clinical guidelines strongly recommend the use of chlorhexidine-impregnated dressings to reduce CRBSI (5, 7). Previously, the main recommendations were focused on: (i) education and training of healthcare personnel who manipulate catheters highlighting hand hygiene; (ii) use of maximal sterile barrier precautions during central venous catheter (CVC) insertion; and (iii) use of >0.5% chlorhexidine skin preparation with alcohol for antisepsis. The scientific evidence generated in the last years has allowed to include in the clinical guidelines the recommendation to use chlorhexidine-impregnated dressings. Specifically, there are three types of commercially available CHG dressings: CHG-impregnated sponge rings, CHG-containing hydrogel pad dressings and dressings with CHG integrated in the adhesive.

Numerous clinical trials have been published assessing CHG dressings performance and safety. The most recent meta-analysis includes 20 studies (18 of them controlled clinical trials) (2). General results show that CHG dressings reduce the risk of CRBSI by 33%, obtaining strongest evidence for adults with short-term CVCs. In contrast, there is a notable risk of contact dermatitis in neonates and pediatric population and lack of evidence of usefulness in these groups. In fact, contact dermatitis was the most common adverse event reported in studies (14, 15). Safdar et al. detected 1.2% of CRBSI in patients receiving CHG dressings compared with 2.3% in patients receiving conventional dressings (14). This study found a significant decreased risk of CRBSI in adult patients admitted in ICUs while no reduction was found in pediatric population. Similarly, another meta-analysis analyzing 12 clinical trials indicate that CHG dressings are useful tools for the prophylaxis of CRBSI (16).

Nevertheless, there are various points to be addressed in the knowledge of CHG dressings. The most important questions are to clarify which groups of patients could obtain a direct benefit from the use of CHG dressings and determine if there are differences among the three types of commercially available CHG dressings (CHG-impregnated sponge rings, CHG-containing hydrogel pad dressings and dressings with CHG integrated in the adhesive) regarding effectivity. Another crucial parameter is the delivery of CHG to the skin and the capacity of absorption of the drug. Some studies have evaluated its absorption in aqueous solutions containing 2% CHG. In an *in vitro* model, topical application showed poor penetration of CHG into the skin (17). In patients, the investigations have focused on newborns and neonates, in which trace amounts of CHG were detected after treating the skins with aqueous solutions (18). The recent advances in the medical field have led to examine the antimicrobial capacity of CHG dressings. A significant reduction of microorganisms was detected along the first week after the placement of the dressing in healthy volunteers (19); however, the quantification of the release pattern of CHG from dressings to the skin has not still been analyzed.

Considering the importance of CHG dressings in the prevention of CRBSI, the aim of this study is to compare the pharmacokinetics and the antimicrobial activity of the novel dressing Oper film® protect CHG to the gold standard product, Tegaderm™ CHG.

## 2 Methods

### 2.1 Materials

Two commercially available chlorhexidine-containing dressings were evaluated and compared in this study: Oper film® protect CHG (Iberhospitex, S.A.) and Tegaderm™ CHG (3 M). Both devices are self-adhesive transparent polyurethane dressings that incorporate a hydrogel that contain 2% CHG pads. These products are indicated to be used in patients eligible for IV catheter placement.

### 2.2 Study population

A total of 25 healthy volunteers were included in the study. Demographic data are shown in Table 1. Subjects were ≥ 18 of age and provided written informed consent. Exclusion criteria were (i) to have incompatibilities with the participation in the study (i.e., to be participating in other clinical trial); (ii) to be allergic/hypersensitive to polyurethane, acrylic adhesive, CHG or any other component of the dressings; (iii) to be pregnant or breastfeeding.

**Table 1:** Demographic data of the patient cohort.

Number of patients	25
Number of dressings	
Oper film® protect CHG	50
Tegaderm™ CHG	50
Mean age	40 ± 12.5 years
Amount of hair	
Absent	23
Some	2
Body sweat	
Absent	28
Some	6
Dryness of the skin	
Absent	18
Some	7

The protocol, conducted in accordance with the GCP standards (CPMP/ICH/135/95) and the current legislation, was approved by the Ethics Committee of Fundació Assitencial Mútua Terrassa. The study was performed at the facilities of Hospital Universitari Mútua Terrassa.

### 2.3 Treatment

**Figure 1:**
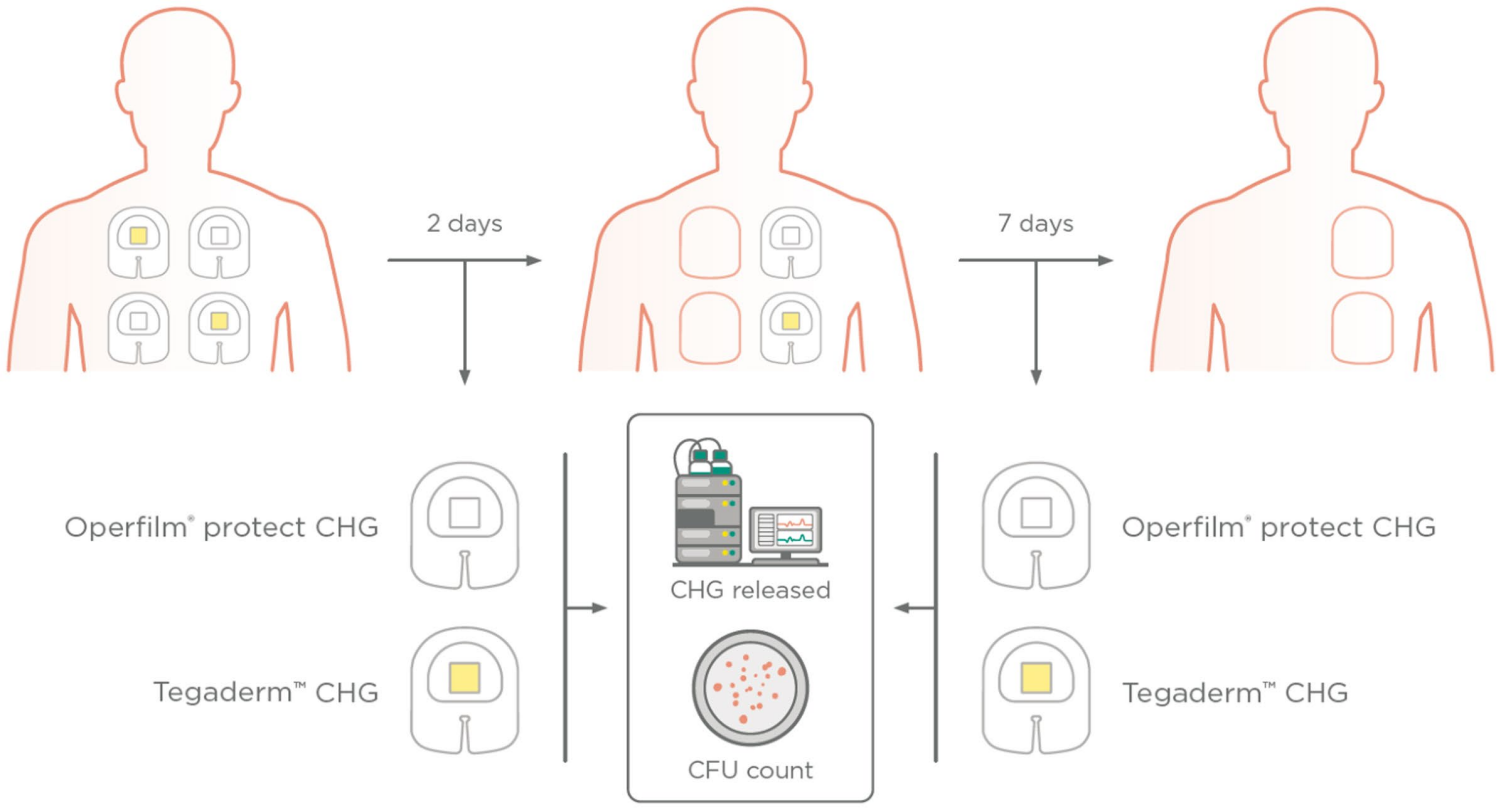
Scheme of the placement of Tegaderm™ CHG and Oper film^®^ protect CHG in the back of the healthy volunteers. The dressings were detached 2 and 7 days after placement. The release of CHG was calculated by HPLC and skin cultures were performed to count the colony forming units (CFU).

Each subject received four dressings on their back. The back, which did not receive disinfection treatment, was divided in two middles (left and right), and each middle contained one Oper film® protect CHG and one Tegaderm™ CHG. The dressings were aleatory allocated in the above or below area (see Figure 1).

Two days after the placement, the two dressings of one of the middles (one Oper film® protect CHG and one Tegaderm™ CHG) were detached and a skin smear from the area covered by the hydrogel pad was immediately taken. Moreover, in order to collect a negative control, a smear from a skin area that had not been in contact with any component of the dressing was collected. All the dressings were stored for subsequent analysis of the quantity of CHG delivered by the hydrogel pad. Seven days after the placement, the two other dressings (one Oper film® protect CHG and one Tegaderm™ CHG) were detached. The same steps described in the previous paragraph were followed.

The subjects could not shower their back during the seven days they wore the dressings. In the case of the appearance of any adverse event, it had to be followed up until it was completely resolved.

### 2.4 Microbial burden

**Figure 2:**
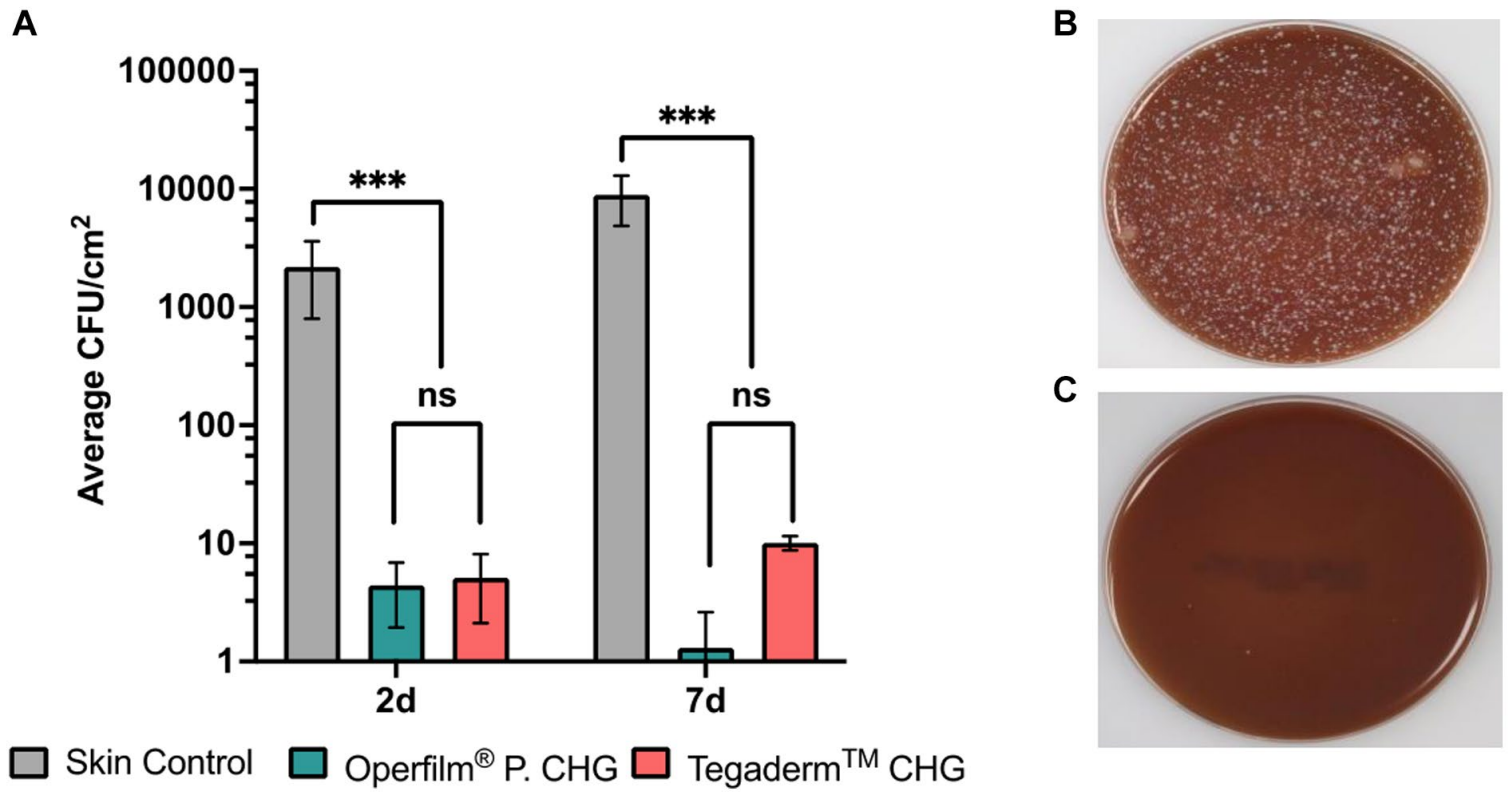
Effect of Oper film^®^ protect CHG and Tegaderm™ CHG on the growth inhibition of microorganisms. **(A)** Colony forming Units (CFU) per square centimeter in skin controls and areas under CHG pads of Tegaderm™ CHG and Oper film^®^ protect CHG. Mean ± SEM (*n* = 25 per group). Groups are compared using Mann–Whitney U non-parametric test; ***p < 0.001, **p < 0.01, *p < 0.05, ns *p* > 0.05. **(B)** representative picture of control skin culture and **(C)** representative picture of a culture from skin treated with Oper film^®^ protect CHG or Tegaderm™ CHG.

The skin under the hydrogel was rubbed in circles with a swab moistened with sterile sodium chloride. The swab was placed in a tube with stuart transport medium (Deltalab, ref. 300291) and stored at 4°C; subsequently, it was expanded on blood agar plates (Scharlab, ref. 064-PA0004) and incubated at 37°C for 48 h. After two days of incubation, a photograph of each plate was taken to count the number of colony-forming units (CFU) grown on the agar. All the area under the hydrogel, that is, all the skin surface that was in contact with CHG, was rubbed with the swab. Thus, the surface indicated in Figure 2 includes the area of the skin that was under the containing-CHG hydrogel. The CFU were manually counted in each culture plate.

### 2.5 CHG quantification

Liquid chromatography was performed on a high-performance liquid chromatography (HPLC) unit from Agilent Technologies 1,200 series. Injections (10 μL) were made on XBridge® C18 column (25 mm × 4.6 mm × 5 μm) from Waters. The column temperature was maintained at 30°C and injector sample racks at 12°C. The flow rate was 1.0 mL/min. The mobile phase was a mix of A: distilled water and acetonitrile (80:20) containing 0.1% TFA and B: distilled water and acetonitrile (10:90) containing 0.1% v/v TFA. The analytical method was validated with respect to parameters such as linearity, range, precision, accuracy, selectivity, and robustness. Chlorhexidine was quantified using an UV–VIS detector. The HPLC method was validated against a Reference Standard from the European Pharmacopoeia (Sigma-Aldrich, ref. PHR1294).

### 2.6 Skin irritation

The erythema degree was evaluated in the skin that was in contact with the chlorhexidine-containing hydrogel. A categorical classification of erythema was elaborated, classifying its degree in absent, mild, moderate or severe. Erythema was monitored on day 2 and day 7 after the placement of the dressing.

### 2.7 Statistical analysis

**Figure 3:**
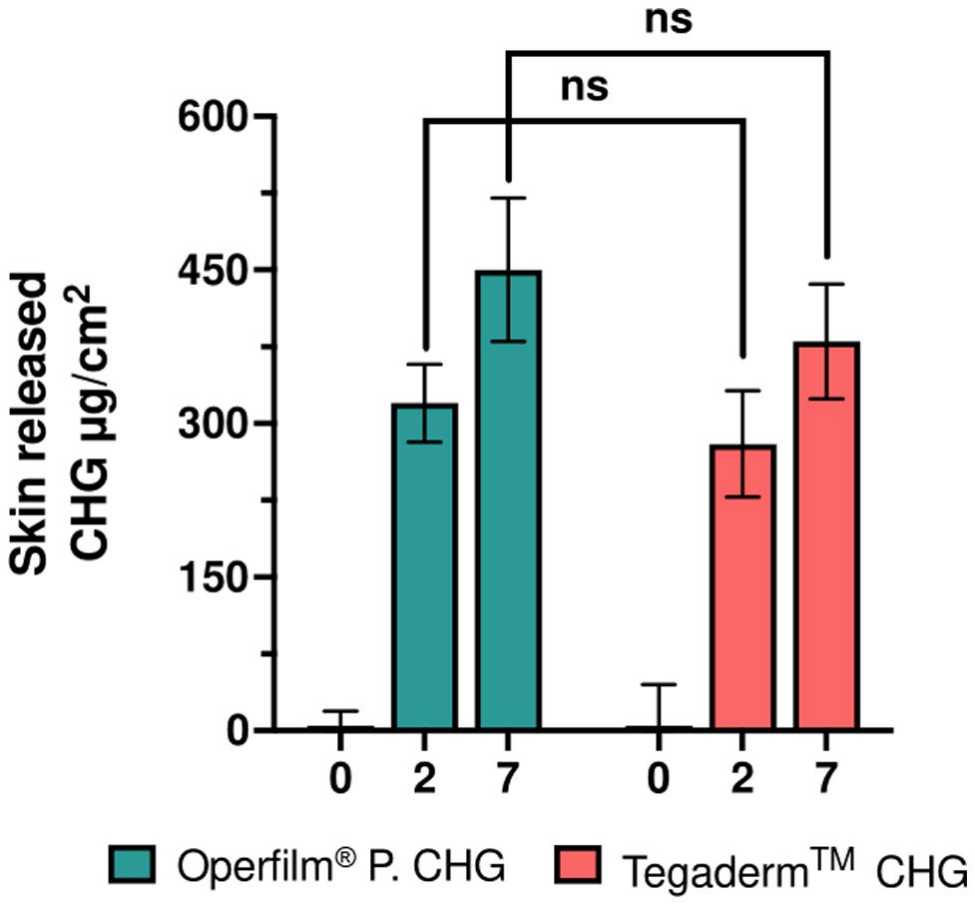
Pharmacokinetics of CHG dressings. CHG delivered to the skin of healthy volunteers at 2 and 7 days after the placement. Mean ± SEM (*n* = 25 per group). Groups are compared using Kruskal-Wallis non-parametric test; ***p < 0.001, **p < 0.01, *p < 0.05, ns *p* > 0.05.

Data is presented as mean ± SEM. Since data did not follow normal distribution, Kruskal-Wallis and Mann–Whitney U non-parametric tests were used to determine differences between groups for CHG release (Figure 3) and microbial count (Figure 2). Fisher’s exact test was used for erythema detection (Table 2). A *p* < 0.05 was considered statistically significant. All statistical analysis and graphical representation were performed using GraphPad Prism.

**Table 2:** Data of log_10_ calculations of cfu/cm^2^ and erythema degree assessment at both time points for each dressing.

	2 days			7 days		
	Control	Oper film^®^ protect CHG	Tegaderm™ CHG	Control	Oper film^®^ protect CHG	Tegaderm™ CHG
*n*	25	25	25	25	25	25
Mean log_10_ cfu/cm^2^	3.34	0.64	0.7	3.95	0.11	1
Standard deviation (log_10_ cfu/cm^2^)	3.84	1.1	1.18	4.3	0.8	0.83
Erythema detection						
Absent	-	24 (96)	22 (88)	-	21 (84)	21 (84)
Low	-	1 (4)	3 (12)	-	4 (16)	4 (16)
Moderate	-	0	0	-	0	0
Severe	-	0	0	-	0	0

## 3 Results

All the 25 volunteers finalized the study. Considering that each subject was applied with 4 dressings (2 Oper film® protect CHG and 2 Tegaderm™ CHG), a total of 100 dressings were used.

The quantity of CHG that remained in the hydrogel was measured by HPLC. Thus, the absorption of the drug was inferred from these values. Two days after the placement of the dressings, Oper film® protect CHG (321 μg/cm^2^) released an equivalent amount of CHG compared to Tegaderm™ CHG (279 μg/cm^2^). Similar results were obtained at 7 days (Oper film® protect CHG, 456 μg/cm^2^; Tegaderm™ CHG, 381 μg/cm^2^) since no significant differences were detected in this pharmacokinetic parameter between the two products (Figure 3). The detected CHG values could be affected by a range of factors such as sweating, the type of skin or the type of patient. These characteristics could notably influence the absolute quantified values; however, the impact in the relative differences between groups or temporal points would be limited.

The count of CFU revealed that both Oper film® protect CHG and Tegaderm™ CHG dramatically reduced the microbial burden at 2 days as well as at 7 days after the placement of the dressings. Moreover, the differences in the decrease of CFU between the two dressings were not significant at neither time point (Figure 2A). These results are shown in detail in Table 2. No group of dressings had a count that exceeded 1 log_10_ cfu/cm^2^. Pictures shown at Figures 2B,C demonstrate an equivalent 2/3-fold log reduction in CFU count produced by the two products. The skin of the patients was not disinfected, thus the microorganisms’ levels of the skin remained intact. Therefore, the non-disinfection of the skin would be the worst situation in which these dressings would be used, and the results demonstrate that under these conditions Oper film® protect CHG possesses a strong antimicrobial activity.

Erythema degree was evaluated in the skin surface that was in contact with the chlorhexidine-containing hydrogel to assess if one week of permanent contact with the drug leads to irritation. Table 2 lists the number and percentages of subjects in each grade of erythema at both clinical visits. At day 2, only 1 volunteer treated with Oper film® protect CHG and 3 volunteers that received Tegaderm™ CHG had low erythema detection. In the second and last visit, low erythema was observed in 4 subjects of each group. No statistical differences were detected neither at 2 days (*p* = 0.6) nor at 7 days (*p* > 0.99). These results confirm the good tolerability of released CHG by these dressings to adult human skin since, after 1 week of permanent contact, 84% of the cohort did not suffer erythema; furthermore, the affected volunteers had a low skin reaction. Moderate or severe erythema was not detected in any subject.

## 4 Discussion

In this study, we have demonstrated that the novel dressing Oper film® protect CHG possesses equivalent CHG release pattern and antimicrobial activity to Tegaderm™ CHG. Furthermore, the results determine that Oper film® protect CHG maintains the antimicrobial action during at least 7 days (Figure 2), which is the maximum period of time the dressing is indicated to be in contact with the skin. Other studies have analyzed the antimicrobial capacity of gels and sponges containing CHG in healthy volunteers during the first week after application; specifically, these investigations studied 3 time points, 1 day, 4 days, and 7 days post-placement of the dressing (19, 20). The results obtained in these studies are similar to the values we show at Figure 2 and confirm the efficiency of the dressings along the week (19, 20).

Baseline log counts detected between 3 and 3.5 log_10_ cfu/cm^2^ (19, 20); in our case, we have detected between 3.3 and 3.9 log_10_ cfu/cm^2^ (Figure 2). Bashir et al. demonstrated that both CHG-containing gel (Tegaderm™ CHG) and disk (Biopatch™ CHG) significantly reduced the microbial count, and similar performance were seen between both types of dressings (19). However, in this study antisepsis was applied using a commercially available skin solution that contained 2% CHG in 70% isopropyl alcohol, which substantially decrease the number of CFU when dressings are placed. In the present work, we showed the results obtained in non-disinfected skin prior to dressing application. We have observed a dramatic and equivalent decline in CFU counts for both hydrogel-based dressings and at both time points, reaching values between 0 and 1 log10 cfu/cm^2^ (Figure 2; Table 2). Although this is the worst situation in which the dressings could be used, the inhibition capacity is very similar to the obtained with disinfected skin (19), finding that demonstrates the strong antimicrobial capacity of Oper film® protect CHG.

The antimicrobial capacity of dressings that integrate CHG in the adhesive have also been studied in healthy volunteers (20). The results indicate a lower antimicrobial activity of this type of dressings compared to CHG-containing hydrogel dressings. This study shows around 2–2.5 log_10_ cfu/cm^2^ at three time points (1, 4, and 7 days after the placement) while we have obtained values between 0 and 1 for both dressing in all the measurements (Table 2). Hence, these results indicate that, at least in healthy skin, not all the dressings that contain CHG have the same performance.

The good antimicrobial activity is related with the release pattern of CHG. Nonetheless, the delivery of CHG from the dressing into the skin have been poorly investigated. One *in vitro* study evaluated the kinetics of delivery of CHG-containing sponges, which detected an increasing CHG concentration in saline medium (21). Here, we show the quantification of the drug in two hydrogel-based dressings. The delivery of CHG is progressively increased throughout the week, which demonstrates that the hydrogel enables a sustained and continued release of CHG. In addition, the amount of CHG transferred to the skin is equivalent between both Oper film® protect CHG and Tegaderm™ CHG (Figure 3). These results are aligned and correlate with the inhibition of microorganisms shown in Figure 2. Therefore, a prolonged and sustained release of CHG into the skin for one week affords a powerful antimicrobial response during all this time.

This continued and prolonged inhibition of microbial growth is the cause of the reduction of CRBSI incidence by CHG-containing dressings, a clinical benefit extensively reported in the literature. Three meta-analyses have concluded that the use of CHG dressings provide significant reduction of the risk of catheter colonization and CRBSI in adult patients with central venous catheters compared to traditional dressings used to protect the insertion site (2, 14, 16). Besides dressings, CHG has also been used in the coating of CVCs as an antibiofilm agent due to the inherent ability of several bacterial and fungi to form biofilms that enable them to evade the host immune response (6, 22).

Contact dermatitis is the most common adverse effect reported in clinical trials (14). As it is known that CHG is the causal agent of this adverse event (2, 23), it was explored the dermal reaction in the area under the hydrogel, which is the part of the dressing that contains and delivers the drug.

No statistical differences in skin irritation were detected between dressing groups, albeit Tegaderm™ CHG may have a slightly higher tendency to irritate. Some scientific publications have detected medical adhesive related skin injury (MARSI) associated to the use of Tegaderm™ CHG (8, 10). Adhesion is mainly based on a balance between mechanical damage to the skin and the ability to avoid dressing detachment, and this equilibrium and clinical benefit must be clinically stablished.

We did not detect any major skin reaction, and only low erythema degree was observed in few patients (Table 2). Although the drug was continuously released during one week, only 16% of volunteers experienced low erythema, which confirms the good safety profile of CHG-containing hydrogel dressings while maintaining the microbial inhibition properties (Table 2).

Currently, there are three main types of CHG dressings commercially available: CHG-impregnated sponge rings, CHG-containing hydrogel pad dressings and dressings with CHG integrated in the adhesive (11, 12, 14). Specifically, hydrogel pads present some advantages compared to sponge rings, such as Biopatch™ CHG, due to improved visibility of the insertion point of the catheter and homogenous CHG release (15). Moreover, dressing disruptions are less frequent in gel dressings, probably explained by the difficulty (24). Even though it is not confirmed, dressing disruption could be a risk factor for CRBSI and thus should be prevented. On the other hand, the antimicrobial capacity of dressings that contain CHG in the adhesive seems to be inferior (20), and is necessary to provide evidence to clarify if this type of dressings reduce CRBSI to a comparable level to gel-based dressings.

Another important factor is the pH of the skin. The acid pH of the skin, that ranges from 4.1 to 5.8, is a key component to maintain a healthy skin since acidic environments inhibit microorganisms’ growth and avoid bacterial colonization (16). The unique formulation of Oper film® protect CHG buffers skin pH and consequently limits the potential infection development. This technical attribute may provide an additional advantage in preventing CRBSI.

## 5 Conclusion

The novel dressing Oper film® protect CHG possesses equivalent CHG release pattern and antimicrobial activity to Tegaderm™ CHG, the gold standard product among CHG dressings. Furthermore, the results of this study determine that Oper film® protect CHG maintains the release of CHG and the antimicrobial action during at least 7 days, which is the maximum period of time the dressing is indicated to be in contact with the skin. Moreover, the improved visibility of the insertion point and the management of the surrounding pH provided by Oper film® protect CHG afford an interesting new tool to prevent CRBSI infections.

The study has some limitations. Firstly, it is monocentric, and the sample size is small. This issue was tried to be solved using 4 dressings in each patient to increase the number of units included in the research. Secondly, the cohort is composed by healthy volunteers with a relatively homogenous age. Next clinical studies should be focused on the analysis of microorganism proliferation in the skin of patients admitted to ICUs or hospital specialized services to specifically determine the decline in the rate of CRBSI infections that presents Oper film® protect CHG when used according to its intended purpose.

## Data availability statement

The original contributions presented in the study are included in the article/supplementary material, further inquiries can be directed to the corresponding author.

## Ethics statement

The studies involving humans were approved by Fundació Assistencial Mutua Terrassa. The studies were conducted in accordance with the local legislation and institutional requirements. The participants provided their written informed consent to participate in this study.

## Author contributions

EM: Conceptualization, Formal analysis, Methodology, Project administration, Writing – original draft, Writing – review & editing. LR-R: Methodology, Writing – review & editing. VF-A: Conceptualization, Writing – original draft, Writing – review & editing. SV: Conceptualization, Project administration, Writing – review & editing. F-JA-M: Writing – review & editing. M-DG-C: Writing – review & editing.
